# A construction of a conformal Chebyshev chaotic map based authentication protocol for healthcare telemedicine services

**DOI:** 10.1007/s40747-021-00441-7

**Published:** 2021-06-19

**Authors:** Dharminder Dharminder, Uddeshaya Kumar, Pratik Gupta

**Affiliations:** 1 Department of Mathematics, Amrita School of Engineering, Amrita Vishwa Vidyapeetham, Chennai, India; 2 Department of Mathematics, The LNM Institute of Information Technology, Jaipur, India; 3 Department of Mathematics, Mandsaur University, Mandsaur, India

**Keywords:** Authentication, Security, Privacy, Key agreement, Chaotic mapping

## Abstract

The outbreak of coronavirus has caused widespread global havoc, and the implementation of lockdown to contain the spread of the virus has caused increased levels of online healthcare services. Upgraded network technology gives birth to a new interface “telecare medicine information systems” in short TMIS. In this system, a user from a remote area and a server located at the hospital can establish a connection to share the necessary information between them. But, it is very clear that all the information is always being transmitted over a public channel. Chaotic map possesses a dynamic structure and it plays a very important role in the construction of a secure and efficient authentication protocols, but they are generally found vulnerable to identity-guess, password-guess, impersonation, and stolen smart-card. We have analyzed (Li et al. in Fut Gen Comput Syst 840:149–159, 2018; Madhusudhan and Nayak Chaitanya in A robust authentication scheme for telecare medical information systems, 2008; Zhang et al in Privacy protection for telecare medicine information systems using a chaotic map-based three-factor authenticated key agreement scheme, 2017; Dharminder and Gupta in Pratik security analysis and application of Chebyshev Chaotic map in the authentication protocols, 2019) and found that Bergamo’s attack (IEEE Trans Circ Syst 52(7):1382–1393, 2005) cannot be resisted by the protocol. Although few of the protocols ensures efficient computations but they cannot ensure an anonymous and secure communication. Therefore, we have proposed a secure and efficient chaotic map based authentication protocol that can be used in telecare medicine information system. This protocol supports verified session keys with only two messages of exchange. Moreover, we have analysed the performance of proposed protocol with relevant protocols and it is being implemented in “Automated Validation of Internet Security Protocols and Applications” respectively.

## Introduction

**Fig. 1:**
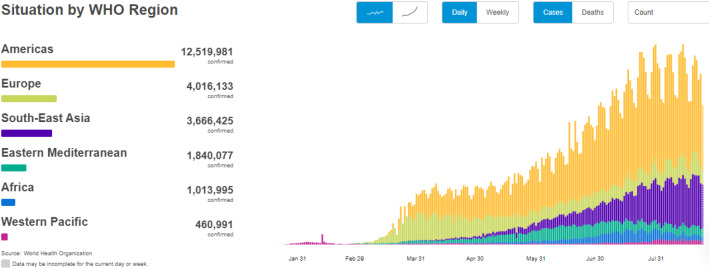
A world wide COVID-19 data recorded on August 25, 2020 by WHO

**Fig. 2:**
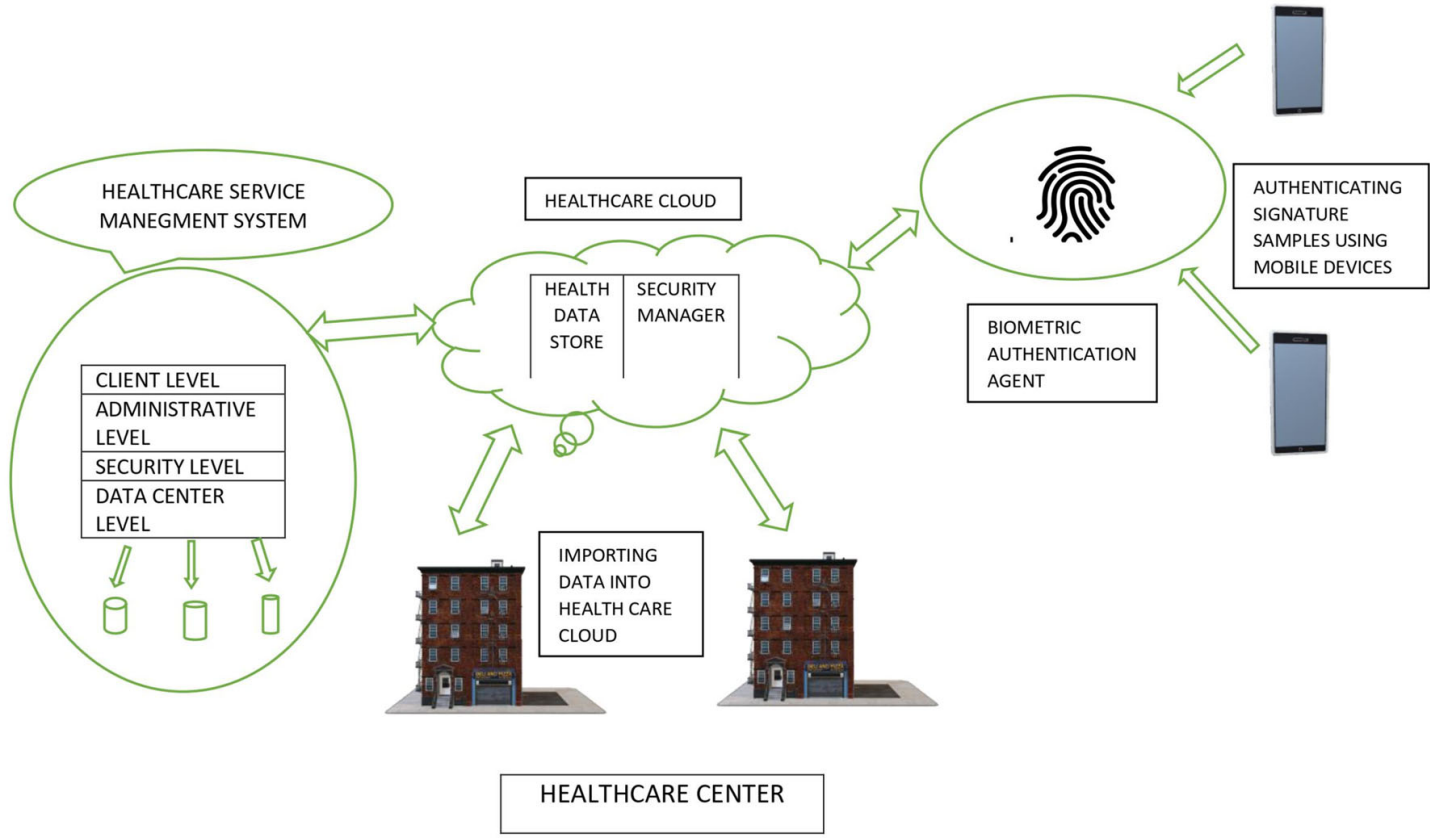
A typical model of health-care services with respect to authentication protocol

The adoption of advanced health care “Telemedicne Information System” and “Telematics” applications in health care needs an integrated strategy to the different social, financial, cultural and political impacts and hurdles of information and communication technologies. Both security and privacy [37–39, 42] are two important attributes required to construct a secure authentication protocol. Security and privacy of “Telemedicne Information System” are the impressive components that are of great interest to the field of health care. Because the Internet is truly an open network with many potential security gaps, close consideration and measures must be required to ensure safe medical facilities and the safety of patient data. Both health care and treatment are two very important factors in the human’s life (see data in Fig. 1). Upgraded technology in the field of online health care services such as variety of medical sensors, smart phones, and smart robotics helps the patients to facilitate the health care services in the remote areas. In these days, most of the doctors are employing robots and smart digital sensor in surgeries is an application of computer science in health care services [40, 41]. There are other applications such as artificial intelligence and machine learning are used to detect the medical conditions of a patient. Nowadays, a patient possessing smart sensors and mobile phones can enjoy benefits of health care services around the world (see Fig. 2).

Patients can be benefited with online health care services via their smart phones, i-pads, and other smart sensors, but their security and privacy are two very important components during communication on public channel. In 2012, Wu et al. [10] designed a secure and anonymous authentication protocol to benefit the patients at their home. In the same year, Wei et al. [9] analyses the security of the protocol [10] and it is found vulnerable to two-factor authentication. In order to eradicate the two-factor authentication defect, a fresh design is needed for two-factor authentication. In the same year, Zhu [12] discussed the security attributes such as password guessing in the protocol [9] and invented a password-guess resistant protocol, although he didn’t seem to think about communicating anonymously. In 2012, Chen et al. [4] designed an efficient and secure lightweight authentication protocol that preserves an anonymous communication in health care telemedicine services.

In 2013, Lin et al.  [7] observed that identity can traced in [4] using both dictionary and password guess along with stolen smart card information. He tried to remove most of the existing attacks and he invented an anonymous authentication protocol. In the same year, Cao and Zhai [3] discussed both security and privacy of [4] and they found that the protocol is vulnerable against both identity guess and password guess along with the information stored in the smart card. Three protocols discussed  [3, 7, 12] are found insecure to input verification procedure due to which they cannot differentiate incorrect inputs with in short time interval. The anonymous communication is another important factor that is missing in  [9, 10, 12, 32] respectively. In 2013, Guo et al. [14] used the complex dynamic structure of chaotic maps to design a new secure authentication protocol, but Hao et al. [15] discussed the security of the protocol and he found that two important attributes traceability and anonymity are missing, and he tried to fill the gap with a new design  [14]. In 2014, Jiang et al. [16] reviewed both security and privacy attributes in [15] and he found the protocol is vulnerable to stolen smart card attack.

In the year 2016, Li et al. [21] designed a secure and efficient chaotic map-based authentication protocol to secure the communication in health care services, but in the year 2018, Madhusudhan et al. [20] discussed the attacks in  [21] such as password guess, and impersonations, and he tried to remove these attacks as discussed in [20]. In the year 2018, Jiang et al. [28] introduced a secure and efficient protocol to improve the telemedicine services in health care sector, but it is not much efficient and it requires to exchange three messages to establish secure and fresh session key. In the same year 2018, Wu et al. [29] introduced a secure and efficient authentication protocol based on RFID and Radhakrishnan et al. [19] also proposed a new design to secure the health care telemedicine services, but their protocols found susceptible to password guess, identity guess and also for stolen card information too. In the same year 2018, Zhang et al. [25] introduced a lightweight and secure authentication protocol for the mobile devices used in heath care telemedicine services, but it is also susceptible to identity guess, password guess and replay attacks. In 2018, Madhusudhan et al. [20] designed an efficient, and secure, and robust protocol for telecare services, but Dharminder et al.  [35] discussed the security of the protocol [20] and they found it susceptible to identity guess, password guess, impersonations, and stolen smart card. In the same year 2020, Dharminder et al. [34] introduced a new design for authentication scheme based on RSA, but it uses the modulo operations that decreases the efficiency of the protocol due to costly modulo exponentiation.

**Table 1:** A review on security attributes of authentication protocols based on password for TMIS

Security parameters$\setminus$protocols	[3]	[4]	[11]	[7]	[30]	[24]	[13]	[31]
Anonymity	$\surd$	$\surd$	$\surd$	$\surd$	$\surd$	$\surd$	$\surd$	$\times$
Password-guess	$\times$	$\times$	$\times$	$\surd$	$\surd$	$\surd$	$\times$	$\times$
Session-key verification	$\surd$	$\times$	$\times$	$\times$	$\times$	$\surd$	$\times$	$\times$
Impersonations	$\surd$	$\times$	$\surd$	$\surd$	$\surd$	$\surd$	$\times$	$\surd$
Replay-messages	$\times$	$\surd$	$\surd$	$\surd$	$\surd$	$\surd$	$\surd$	$\surd$
Key-agreement	$\surd$	$\surd$	$\surd$	$\surd$	$\surd$	$\surd$	$\surd$	$\surd$
Stolen-card	$\surd$	$\surd$	$\surd$	$\surd$	$\surd$	$\surd$	$\surd$	$\surd$
Password-change	$\surd$	$\surd$	$\times$	$\times$	$\times$	$\times$	$\times$	$\surd$

**Table 2:** A review of security attributes of authentication protocols based on chaotic map for TMIS

Security parameters/protocols	[24]	[15]	[22]	[23]	[19]	[25]	[21]	[20]
Anonymity	$\surd$	$\surd$	$\times$	$\surd$	$\times$	$\times$	$\times$	$\times$
Insider-security	$\surd$	$\surd$	$\surd$	$\surd$	$\surd$	$\surd$	$\surd$	$\surd$
Password-guess	$\surd$	$\surd$	$\surd$	$\surd$	$\times$	$\times$	$\times$	$\times$
Stolen-cards	$\surd$	$\surd$	$\surd$	$\surd$	$\times$	$\surd$	$\surd$	$\times$
User’s impersonations	$\surd$	$\surd$	$\surd$	$\surd$	$\surd$	$\surd$	$\times$	$\times$
Key-agreement	$\surd$	$\surd$	$\surd$	$\surd$	$\surd$	$\surd$	$\surd$	$\surd$
Server’s impersonations	$\surd$	$\surd$	$\surd$	$\surd$	$\surd$	$\surd$	$\times$	$\times$
Replay-messages	$\surd$	$\surd$	$\surd$	$\surd$	$\surd$	$\times$	$\surd$	$\surd$
Key-agreement	$\surd$	$\surd$	$\surd$	$\surd$	$\surd$	$\surd$	$\surd$	$\surd$
Key-verification	$\surd$	$\times$	$\surd$	$\times$	$\surd$	$\surd$	$\times$	$\surd$

In the Table 2, we have observed various security attributes achieved by the existing relevant chaotic map based authentication protocols used to secure TMIS system, where the symbol $\surd$ denotes “yes”, and $\times$ denotes “not” respectively. In the Tables 1, 2 one can see that existing protocols in the TMIS environment suffers various vulnerabilities such as password-guess, identity-guess, impersonations, replaying of older messages, and stolen smart card attacks. In the proposed design, we have discussed two important components security and privacy in the form of security attributes such as identity guess, impersonations, password guess, anonymity, replaying of messages, and stolen smart card information in the protocols [1, 20, 25, 35] respectively. In the design [35], we have analyzed that a user $U_i$ selects $ID_i$, $PW_i$ and calculates $A_i= h(ID_i||PW_i)$, then he sends $<ID_i,~A_i>$ to the server. Next, the server chooses a random $n_i \in Z_p^*$, and does the computation $B_i = T_x(ID_i||n_i)\oplus A_i$, it sends $<h(.),~B_i,~n_i>$ to the corresponding user. Now, the user $U_i$ calculates $B_i\oplus A_i =T_x(ID_i||n_i)$ then $D_i=h(T_x(ID_i||n_i)||PW_i||ID_i)$ and $N_i=n_i \oplus A_i$, and then store $B_i,~D_i,~N_i$ in the corresponding smart card. We have observed that a user knows $n_i \in Z_p^*$, and $T_x(ID_i||n_i)$ can executes Bergamo’s attack and computes $x'$ as $T_{x'}(ID_i||n_i)=T_x(ID_i||n_i)$ and uses $x'$ in the ongoing communication on the open channel.

Similarly, in the design [1], we have analyzed a vulnerability in the session key established during the communication. In the design [1], an adversary $\mathcal {A}$ obtains the information from earlier transmitted information $M_1$ and $M_2$. Moreover, $\mathcal {A}$ computes $u'$ with knowledge of x, $T_{u}(x)$ satisfying $T_{u}(x)=T_{u'}(x)$. Finally, $\mathcal {A}$ guesses $v'$ under the previous knowledge x, $T_v(x)$ satisfies $T_v(x)=T_{v'}(x)$ as:

$$\begin{aligned} u'= & {} \frac{arccos(T_u(x))+2k\pi }{arccos(x)}\\ v'= & {} \frac{arccos(T_v(x))+2k\pi }{arccos(x)} \end{aligned}$$

Where k is a positive integer, finally $\mathcal {A}$ finds $T_{v'} T_{u'}(x)=T_{v} T_{u}(x)=Sk_u=Sk_s$ that plays the role of session key.

Similarly, in the design [25], we have analyzed a vulnerability in the session key established during the communication. In the design [25], an adversary $\mathcal {A}$ obtains the information from earlier transmitted messages $\{a_i,T_u(x),Nid_i \}$, $\{b_i,T_s(x),m\}$ and $\{Y_i,Nid_i,Z_i,h(.),V_i,x \}$ stored in the corresponding smart card. Furthers, $\mathcal {A}$ obtains $u'$ under the knowledge of x, $T_{u}(x)$ satisfies $T_{u}(x)=T_{u'}(x)$. Furthers, $\mathcal {A}$ makes a guess $s'$ under the knowledge of x, $T_s(x)$ satisfies $T_s(x)=T_{s'}(x)$.

$$\begin{aligned} u'= & {} \frac{arccos(T_u(x))+2k\pi }{arccos(x)}\\ s'= & {} \frac{arccos(T_s(x))+2k\pi }{arccos(x)} \end{aligned}$$

Where k is a positive integer, then $\mathcal {A}$ computes $T_{s'}T_{u'}(x)=T_{s}T_{u}(x)=Sk_u=Sk_s$ that plays the role of session key during communication.

Similarly, in the design [20], we have analyzed a vulnerability in the session key established during the communication. In the design [20], an adversary $\mathcal {A}$ obtains the information from earlier transmitted messages $\{Cid_i,E_i,D_i,F_i\}$, $\{H_i,T_z(ID_i||n_i)\}$ and $\{B_i,h(.),C_i,n_i,D_i\}$ that is stored in the smart card. Furthers, $\mathcal {A}$ computes $y'$ under the knowledge $ID_i$, $n_i$, $T_{y}(ID_i||n_i)$ satisfies $T_{y}(ID_i||n_i)=T_{y'}(ID_i||n_i)$. Furthers, $\mathcal {A}$ makes a guess $z'$ under the knowledge $ID_i$, $n_i$, $T_{z}(ID_i||n_i)$ satisfies $T_{z}(ID_i||n_i)=T_{z'}(ID_i||n_i)$ as:

$$\begin{aligned} y'= & {} \frac{arccos(T_u(ID_i||n_i))+2k\pi }{arccos(ID_i||n_i)}\\ z'= & {} \frac{arccos(T_z(ID_i||n_i))+2k\pi }{arccos(ID_i||n_i)} \end{aligned}$$

Where k is a positive integer, then $\mathcal {A}$ computes $T_{s'}T_{u'}(ID_i||n_i)=T_{s}T_{u}(ID_i||n_i)$ and $Sk_i=Sk_j=h(h(ID_i)||T_z'T_y'(ID_i|| n_i)||T_y'(ID_i||n_i))$ that plays the role of session key during communication.

To handle the issues in [1, 20, 25, 35], we have an idea to compute $x =h(ID_i||s)$, where $ID_i$ is the identity of $i^{th}$ user and “s” is the long term secret key of the server, in this way a user possesses *x* that results from different $ID_i$ concatenated with master key of the server to produce different secret keys *x* for each of the user. Now, the *x* will plays the role in place of master secret that is different for each of the user. Therefore, we have designed a new authentication protocol possessing both security and efficiency using the dynamic Chaos theory. The security of the presented scheme have been analyzed in random Oracle with this we also use the tool for authentication called “Automated Validation of Internet Security Protocols and Applications” respectively. Moreover, the presented protocol resists session key violation problem, that is proposed by Bergamo et al. [33] and establishes a session key with just two messages of exchange.

## Preliminaries

In this section, we will discuss some of the basic notations, terminologies and basic properties of conformal Chaos maps used in the proposed protocol. A conformal map is an angle-preserving transformation that preserves local angles. A brief review of some of the useful notations are also given in Table 3.

**Table 3:** Some of useful notation and symbol

Notation	Description
$U_i$	User-i
$Sr_j$	Server-j
$\mathcal {A}$	Adversary
$Sc_i$	Smart Card
$ID_i$	Identity for $U_i$
*TMIS*	Telecare Medicine Information System
$Ps_i$	Password for $U_i$
*s*	Secret Key for $S_j$
$h(\cdot )$	A secure hashing
$bm_i$	biometric imprint
$\oplus$	Bitwise XOR
||	Concatenation of two strings

### Chebyshev chaotic mapping

**Fig. 3:**
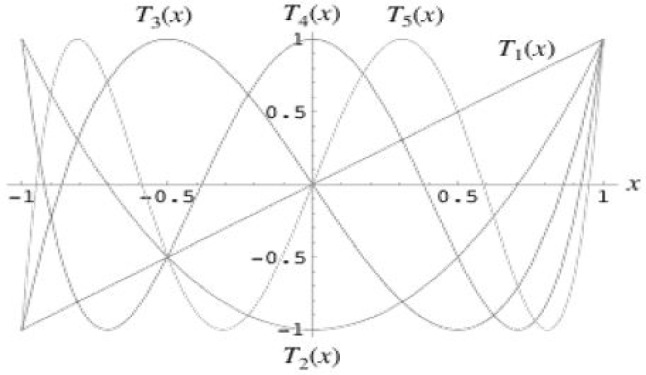
A review of dynamic structure of Chebyshev polynomials

As seen in Fig. 3, chaotic maps have a complex dynamic structure and it is well known for its pseudo randomness. In this subsection, we have discussed some of basic definitions and dynamic properties [17].

#### Definition 1

A polynomial $T_\nu (y):(-\infty ,+\infty )\rightarrow [-1,+1]$ is introduced by Chebyshev in variable “y” and of positive degree $\nu$, besides that $T_\nu (y) = cos(\nu (arccos(y)))$ and the recurrence $T_\nu (y)$ is described as $T_\nu (y) = (2yT_{\nu -1}(y)-T_{\nu -2}(y))$, whereas $y\in (-\infty ,+\infty )$ and $T_0(y) = 1$, $T_1(y) = y$

#### Theorem 1

The set of Chebyshev polynomials on $R=(-\infty ,+\infty )$ satisfies semigroup property under composition of chaotic maps.

#### Proof

Chebyshev polynomials given in variable x is $T_\nu (y):(-\infty ,+\infty )\rightarrow [-1,+1]$, where $\nu >0$ is an integer, and $T_\nu (y) = cos(\nu (arccos(y)))$ under recurrence relation $T_\nu (y) = (2yT_{\nu -1}(y)-T_{\nu -2}(y))$, furthers $y\in (-\infty ,+\infty )$ and $T_0(y) = 1$, $T_1(y) = y$. Therefore, we get difference equation along with characteristics equation $\rho ^2-2y \rho +1=0$. Therefore, $\rho _1=y+\sqrt{y^2-1}$ and $\rho _2=y-\sqrt{y^2-1}$ are two characteristics roots of characteristics equation $\rho ^2-2y \rho +1=0$ along with $\rho _1+\rho _2=2y$ and $\rho _1 \rho _2=1$. Therefore,

$$\begin{aligned} T_n(y)= & {} \frac{\rho _1^n+\rho _2^n}{2} \\= & {} \frac{(y+\sqrt{y^2-1})^n+(y-\sqrt{y^2-1})^n}{2} \\= & {} \sum _{i=0}^{[\frac{n}{2}]} C_i^n y^{n-2i}(y^2-1)^i \end{aligned}$$

Where n, i are positive integers and $C_i^n=\frac{n!}{i!(n-i)!}$. Furthers, $T_{n_1}(T_{n_2}(y))=\frac{\xi _1^{n_1}+ \xi _2^{n_1}}{2}$ where $n_1$, $n_2$ are positive integers and $\xi _1+\xi _2=2 T_{n_2}(y)$, and $\xi _1 \xi _2=1$. Therefore, $\xi _1+\xi _2^{-1}=\rho _1^{n_2}+\rho _1^{-n_2}$ then $\xi _1=\rho _1^{n_2}$ or $\xi _1=\rho _1^{-n_2}$. Furthermore, $T_n(y) = \frac{\rho _1^n+\rho _2^n}{2}$ gives us $T_{n_1 n_2}(y) = \frac{\rho _1^{n_1 n_2}+\rho _2^{n_1 n_2}}{2}$. Hence $T_{n_1 n_2}(y)= T_{n_1}(T_{n_2}(y))= T_{n_2}(T_{n_1}(y))= T_{n_2 n_1}(y)$. $\square$

#### Definition 2

If it is asked to find *u* such that $T_u(x) = y$, where the values *y* and *x* are known to the adversary. Then this problem is know as Discrete Logarithm Problem (DLP).

#### Definition 3

Computational Diffie-Hellman Problem (CDHP) can be stated to find $$T_{uv}(x)$$, where the values $$x,~T_u(x)$$ and $$T_v(x) $$ are known to the adversary.

### Fuzzy extractor

A fuzzy extractor($E_f$) [2] is an extraction mechanism that is used to extract a random uniform string from biometric imprints ($bm_i$). It consists of two algorithms *I*(.) and *R*(.). *I*(.) is a probabilistic algorithm that produced two strings $b_1,~b_2$ as output after taking $bm_i$ an input parameter, where $b_1$ is private key and $b_2$ is a helper string. *R*(.) is an algorithm that is used to regenerate the private key $b_1$ after taking noisy biometric parameter $bm_i'$ and helper string $b_2$ as input, where $|bm_i-bm_i'|\le \delta bm$.

## Proposed authentication protocol under chaotic mapping

We have proposed a secure and efficient chaotic map based authentication that can be divided into four phases, (1) registration-phase, (2) login-phase, (3) authentication-phase and (4) password-update-phase.

### Registration-phase

$U_i$ registers to the concern server $Sr_j$ via a secure channel as written in the following lines.

- $U_i$ selects $ID_i$, $Ps_i$, and imprints his own biometric $bm_i$ then compute $I(bm_i)=(b_1,b_2)$, $Ui_1= h(ID_i||Ps_i||b_1)$ and transmits $\{ID_i,~Ui_1\}$ to $Sr_j$.
- After getting the information $\{ID_i,~Ui_1\}$, $Sr_j$ chooses arbitrary $p_i \in Z_p^*$, then compute first $x=h(ID_i||s)$, where “s” is the private key of $Sr_j$. Now, it computes $Si_1=T_s(ID_i)$, $Si_2=T_x(ID||p_i)$. Then, it calculates the value $Si_3 = (Si_1||Si_2)\oplus Ui_1$ and store $h(.),~Si_3,~p_i$.
- $Sr_j$ delivers a hidden information $\{h(.),~Si_3,~p_i\}$, after stored it into the smart-card, to $U_i$ via a private channel.
- Finally, $U_i$ does the computations $Si_1||Si_2=Si_3 \oplus Ui_1 ,~Ui_2=h(Si_1 \Vert Si_2||Ps_i||ID_i||b_1)$, $P_i=p_i \oplus Ui_1$ and store $\{ h(.),~Si_3,~Ui_2,~P_i,~b_2 \}$ into the smart card.

**Fig. 4:**
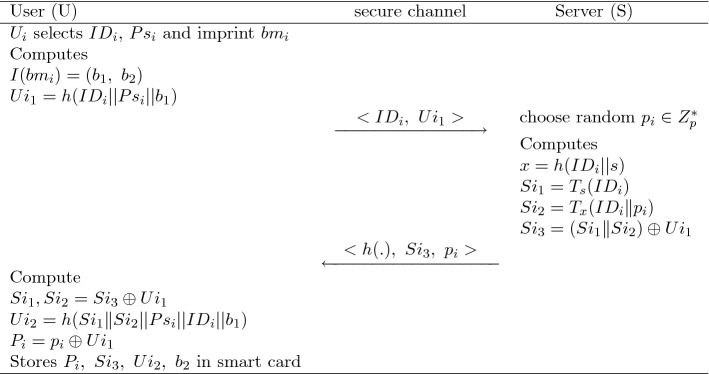
A description of registration phase via secure channel

**Fig. 5:**
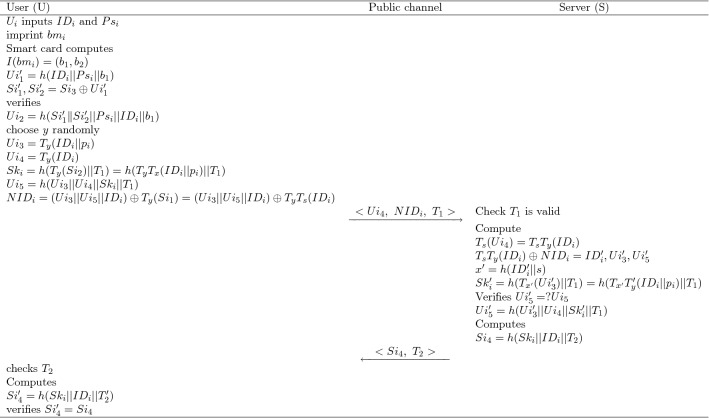
A description of of login and authentication phases

### Login phase

If $U_i$ wants to login to $Sr_j$ then:

- $U_i$ firstly insert the card into card reader machine, then input $ID_i$, $Ps_i$ and imprints the biometric $bm_i$, then the card computes $I(bm_i)=(b_1,~b_2)$ and $Ui_1'= h(ID_i||Ps_i||b_1)$.
- Using $Ui_1'$ smart card executes the step $Si_1' ,Si_2'=Si_3\oplus Ui_1'$ and computes $Ui_2'=h(Si_1' \Vert Si_2'||Ps_i||ID_i||b_1)$ and proceeds for the legal verification $Ui_2'=?Ui_2$.
- The choose $y \in Z_p^*$ arbitrarily and furthers proceeds to compute $Ui_3=T_y(ID_i||p_i)$, $Ui_4=T_y(ID_i)$, $Sk_i=h(T_y(Si_2)||T_1)=h(T_yT_x(ID_i||p_i)||T_1)$, $Ui_5=h(Ui_3||Ui_4||Sk_i||T_1)$, $NID_i=(Ui_3||Ui_5||ID_i) \oplus T_y (Si_1)=(Ui_3||Ui_5||ID_i \oplus T_y T_s(ID_i)$ and then $U_i$ transmits ${~<Ui_4, ~NID_i,~T_1>~}$ to $Sr_j$.

### Authentication phase

$Sr_j$ receives ${~<Ui_4, ~NID_i,~T_1>~}$ from corresponding $U_i$ and executes the process of authentication:

- $Sr_j$ verifies the time stamp $T_1$, and computes $T_s(Ui_4)=T_sT_y(ID_i)$, $T_sT_y(ID_i) \oplus NID_i=ID_i',Ui_3',Ui_5'$ then after getting $ID_i'$ $Sr_j$ computes $x'=h(ID_i'||s)$, $Sk_i'=h(T_{x'}(Ui_3')||T_1)=h(T_{x'}T_{y}'(ID_i||p_i)||T_1)$ and then verifies $Ui_5'=?Ui_5$ where $Ui_5'=h(Ui_3'||Ui_4||Sk_i'||T_1)$ finally $Sr_j$ computes $Si_4=h(Sk_i||ID_i||T_2)$.
- $Sr_j$ transmits the information $\{Si_4,~T_2\}$ to $U_i$ through public channel.
- After receiving the information $$\{Si_4,~T_2\}$$ from $$Sr_j$$, then $$U_i$$ confirms $$T_2$$ is valid or not, then he calculates $$Si_4'=h(Sk_i||ID_i||T_2)$$, and proceeds the verification $$ Si_4'=Si_4$$, and establishes a session key $$Sk_i=h(T_yT_x(ID_i||p_i)||T_1)$$.

### Password update phase

To update password $U_i$, executes the following steps:

- $U_i$ inputs $ID_i$ and $Ps_i$. Furthers, he imprints biometric, then computes $I(bm_i)=(b_1,~b_2)$ and $Ui_1'= h(ID_i||Ps_i||b_1)$. Using $Ui_1'$, the $Sc_i$ obtains $Si_1' ,Si_2'=Si_3\oplus Ui_1'$ and $Ui_2'=h(Si_1' \Vert Si_2'||Ps_i||ID_i||b_1)$ and proceeds for the verification $Ui_2'=Ui_2$.
- $$U_i$$ inputs $$Ps_i^{new}$$ and proceeds the computation $$Ui_1^{new}= h(ID_i||Ps_i^{new}||b_1)$$, $$Ui_2^{new}=h(Si_1'||Si_2'||Ps_{i}^{new}||ID_i||b_1)$$, $$Si_3^{new}=Ui_1^{new} \oplus Si_1||Si_2 $$ and updates the values $$Si_3,~Ui_2$$ with $$ Si_3^{new},~ Ui_2^{new}$$.

## Security analysis

### Formal security analysis

At first, we have to define a framework $\mathcal {P}$ to verify the security of the presented protocol and then, under random oracle, we will implement the presented protocol.

**Security-model** Suppose the $i^{th}$ instance of a user $U_i$ is denoted by $M_i \in (U_i,~Sr_j)$, and $\mathcal {A}$ be an attacker that governs the connection between $U_i$ and $Sr_j$. An illustration of $\mathcal {A}$, is therefore stated as follows:

**Extract**: With the help of extract query, $\mathcal {A}$ could get the private key of a user $U_i$.

**Send**$(m, M_i)$: With the help of send query, $\mathcal {A}$ could be able to send arbitrary message *m*, to random oracle then in response of *m*, random oracle have to reply with a computational output.

**Hash(m):** In this query $\mathcal {A}$ sends random massage *m* to *H*(.), then oracle select $s \in Z_p^*$ randomly and reply with *s*, after storing it into hash list $H_{ij}$ with *m*. Initially $H_{ij}$ assumed to be empty.

**Reveal**$(M_i)$: If an adversary $\mathcal {A}$ process a reveal query to oracle, then oracle permits $\mathcal {A}$, to know about session key $Sk_i$.

**Corrupt**$(M_i)$: Corrupt query allows $\mathcal {A}$ to obtains the private key of $M_i$, by corrupting $M_i$.

**Test**$(M_i)$: If oracle receives test query, oracle guesses a random bit $b \in \{0,1\}$. Then two cases aries: (1) if b = 0, then oracle reply with arbitrary number, (2) if b = 1, then oracle reply with session key $Sk_{i}$.

Suppose a bit b is selected from corrupt query phase and $Succ(\mathcal {A})$ correctly estimates the value of b, then the advantage $Adv_{\mathcal {A},P}(k)$, against the protocol retained by adversary is specified as:

$$\begin{aligned} Adv_{\mathcal {A},P}(k) = |2 . Pr[Succ(\mathcal {A})]-1| \end{aligned}$$

Mutual authentication is established by security analysis in Random Oracle for the suggested scheme.

**Chaotic based assumption:** Suppose $x \in Z_p^*$ is a secret key of $Sr_j$, p is a prime number with length n, then from generation algorithm $Gen(1^{n})$ = *p*

$\exists$ a negligible function *neg*(*n*) such that:

$$\begin{aligned}&Pr[Gen(1^{n})\rightarrow p,x,r,T_x(r) \leftarrow {Z_p}* \\&:\mathcal {A} (1^{n},p,T_x(r))\rightarrow x] = neg(n) \end{aligned}$$

$\forall$ probabilistic polynomial time adversary $\mathcal {A}$.

**Collision resistance attack(CRA) algorithm:** If for *h*(.), $\mathcal {A}$, then we have

$$\begin{aligned} Adv_\mathcal {A} = Pr[(c,c') \overset{R}{\leftarrow } Adv:c\ne c' and h(c)=h(c')] \end{aligned}$$

#### Theorem 2

Suppose $\mathcal {R}$ be a RO and if $\mathcal {A}$ breach the suggested mutual authentication protocol, then we can design an algorithm $M_o$, to solve *CRA* problem, together with $\mathcal {A}$.

#### Proof

At first, $M_o$ detects $Ui_4,~NID_i,~H_i$ and then to solve the problem of *CRA*, $M_o$ attempts to calculate ${X_{i}}*$ and ${r}*$ and verifies $h(r^*||Ps_i||ID_i||H_i) = ? Ui_2$ or $Ui_5=?h(Ui_3||Ui_4||Sk_u||T_1))$. $M_o$ have access to $\mathcal {R},,~p,~\omega ,~T,~Gen(.)$, also $M_o$ can reach to $\mathcal {A}$ through query. $\square$

**Hash query:** When $\mathcal {A}$ asks hash query $H_q$, for $ID_i$, $M_o$ first check $H_{ij}$ list for $ID_i$. If $M_o$ found it in the list $H_{ij}$, then it reply $h_{i1}$, otherwise $M_o$ calculates $h_u = H_1(ID_i)$ and put it in the list along with $ID_i$, and sends $h_u$ back to $\mathcal {A}$.

**Extract:** In extract query $$\mathcal {A}$$ sends query on $$ID_i$$. After getting extract query $$M_o$$ checks $$h_u\in \{ H_i, Ui_4,NID_i\}$$. If $$M_o$$ fails to verify, then $$M_o$$ ends the process. After this, $$M_o$$ searches $$ID_i \in H_{ij}$$, if present, then it responds, else calculates $$X_i = Ui_4$$ and return $$h(Si_2||Ps_i||ID_i||H_i) = Ui_2 $$ to $$\mathcal {A}$$.

**Send-queries:** In send query there are three phases discussed as below, first $U_i$ request for login to $Sr_j$, then $U_i$ sends a message $<(Ui_4,~NID_i,~T_1)>$ to $Sr_j$, and at last $Sr_j$ responds $(Si_4,T_2)$. We will describe this phase by a game between $U_i$ and $Sr_j$ respectively.

1. $\mathcal {A}$ start it with sending a query, in response of that *Mo* is supposed to reply a login message to $\mathcal {A}$.
2. $\mathcal {A}$ sends polynomial time send queries, to login into $Sr_j$ then $M_o$ computes corresponding to $i^{th}$ query as $Ui_4 = T_y(ID_i)$ and $Ui_2= h(Si_2||Ps_i||ID_i||p_i)$ responds to $\mathcal {A}$.
3. $\mathcal {A}$ submits $(Ui_2,~U_i)$, then $M_o$ verifies first whether $h_u\in H_{ij}$ or not. If it is present, then $M_o$ returns a failure, else ends the query ${Q_{e_1}}$. Furthers, *Mo* computes $R_1 = T_y(ID_i||p_i)$, and $Sk=T_jT_y(ID_i||p_i)$ for arbitrary *j*, *y* and $E_i=h(T_y(ID_i||p_i)||Sk||T_1)$ and returns the output to $\mathcal {A}$.
4. $\mathcal {A}$ submits $((T_y(r_i),Ui_5),LS_j)$, then $M_o$ computes $S_i = h(Id_i||r)$ and $Ui_2= h(T_x(ID_i||p_i)||Ps_i||ID_i||p_i)$, where *r* is chosen arbitrarily, then check the equation $W_i =? h(T_x(r_i)||Ps_i||ID_i||p_i)$, if holds, then $M_o$ does the computation $Sk = T_yT_j(ID_i||N_i)$, $Si_4 = h(Sk||T_2)$ responds $(Si_4, T_2)$ to $\mathcal {A}$.
5. $\mathcal {A}$ submits $((Si_4,T_2),U_i)$, then $M_o$ does computations discussed above and get $V_2$, then verify ${Si_4}* =? h(Sk||T_2)$ and at last authenticates $\mathcal {A}$.

Analysis: If $\mathcal {A}$ violates the authentication process between $U_i$ and $Sr_j$, then without knowing about the private key, the authentication massage $M_1=T_y(Si_2)$ could be forged by $\mathcal {A}$. $\mathcal {A}$ can send a duplicate massage ${Si_i}*$

If without any knowledge about privet key, $\mathcal {A}$ is able to forge $M_1=T_y(r_i)$, then it can sends a duplicate ${Si_i}*$. If $M_1=h(X)\in L_{ij}$, then failed to proceed $P_{c_2}$. Else, $M_o$ solves the problem of *CRA*. As $H(ID_i) \not \in L_{ij}$ , $A=h(B) \not \in (s_{i1},s_{i2} \dots )$, and let the chance of success of $M_o$ be $\alpha$, and the chance of violating the protocol be $\beta$ respectively. Then, if event $P_c$ , $P_{c_1}$, $P_{c_2}$ exist then hash query, extract query and send query are legal. So, to break *CRA* problem $M_o$ takes the help of $\mathcal {A}$, if none of $P_c$ , $P_{c_1}$, $P_{c_2}$ happened. Thus,

$$\begin{aligned} Pr[\lnot P_c \wedge \lnot P_{c_1} \wedge \lnot P_{c_2}] = \left( \frac{P_q}{H_q}\right) ^{P_q +S_q } \left( \frac{H_{q}-E_q}{H_q}\right) \end{aligned}$$

Therefore, $M_o$ is successful with advantage

$$\alpha \ge \left( \beta - \frac{1}{2^{k}}\right) \left( \frac{P_q}{H_q}\right) ^{P_q +s_q } \left( \frac{H_{q}-P_q}{H_q}\right) $$

So, we can observe that the algorithm $M_o$ has the advantage, so if $\mathcal {A}$ can breach the scheme, then $M_o$ can breach the suggested protocol, using subroutine $\mathcal {A}$.

#### Theorem 3

The protocol is secured against chosen massage attack in RO model, if Chaotic discrete logarithm problem (CDLP) holds.

#### Proof

By contradiction, let us prove this, let us say that there is $\mathcal {A}$, who breaches proposed scheme against chosen massage attack, so we can model model an algorithm *mo* that violates the discrete logarithm presumption based on the Chaotic map (CDLP), which implies that $\mathcal {A}$ breaches the proposed system, then *mo* breaches the proposed system as well, which means $\mathcal {A}$ breaches the proposed scheme with non-negligible advantage.

- Game 1: Suppose that $\mathcal {A}$ is playing a chosen massage attack game with the suggested scheme. At first, $\mathcal {A}$ possesses security parameter $1^{n}$ and map $\mathcal {T}$, then $\mathcal {A}$ use massage space $\mathcal {M}$, to send massages $(M_0, M_1)$, to perform encryption queries $Q_E$. After getting massage, oracle selects arbitrary $m\in \{0, 1\}$ and applies $E_1 = \mathcal {T}_x(M_m)$ and selects $y\in \{0, 1\}^{n}$ computes $E_2 = y \oplus M_m$, then reply $E_m = (E_1 , E_2)$. Finally, if $\mathcal {A}$ guesses a correct bit $m'$, where $m = m'$ then $\mathcal {A}$ wins the game.
- Game 2: In this phase *Mo* tries to breach the assumptions. Since CDLP says challenger can obtain $s, q\leftarrow {Z_n}*$ by running *Gen*(.) algorithm and returns ($1^n , \mathcal {T}_y(s)$ ) to *Mo*. Now *Mo* has to return s without having y.
- Game 3: To guess *y* correctly, $\mathcal {A}$ has three options:
  1. If $\mathcal {T}_z\mathcal {T}_x(r) = \mathcal {T}_{z'}\mathcal {T}_x(r)$ implies $z'=z$, $\mathcal {A}$ continues to oracle with a random z, then it calculates $C_1 = \mathcal {T}_z(r)$, else $RO(z) = y$, and reports $y'= y$. However, $\mathcal {A}$ have non negligible chance of conducting such a query, so *Mo* will win with a non-negligible gain. However, it has been concluded that *Mo* violates the assumption with a marginal chance that implies $\mathcal {A}$ has a neg(n) gain.
  2. In this case $\mathcal {A}$ have no clue about *y* because there is no query about *z* following $\mathcal {T}_z\mathcal {T}_x(r) = \mathcal {T}_{z'}\mathcal {T}_x(r)$. Now since $C_2 = y\oplus M_b$ works as one time pad so the probability of winning of $\mathcal {A}$ is $Pr[\mathcal {A}: win ] = \frac{1}{2}$.
  3. So the probability of $\mathcal {A}$ for winning the game is $Pr[\mathcal {A}: win] = \frac{1}{2}+neg(n)$, that contradicts our assumption.

$$\square $$

### Informal security analysis

#### Anonymous

If for any protocol, it is impossible for any adversary, to find user’s real identity then we says that protocol follows the anonymous property. In our protocol, user’s identity is not used over public channel, instead we use $NID_i=(Ui_3||Ui_5||ID_i)\oplus T_y(Si_1)$. Adversary can intercept $NID_i$ and $Ui_4$ but it is not possible for adversary to extract $ID_i$ from $NID_i$ because for that $\mathcal {A}$ needs to compute $T_s(Ui_4)$ that requires long term secret key *s* of server.

#### Password guessing attack

Password guessing attack is not possible on proposed scheme because $\mathcal {A}$ needs $Si_3,~Ui_2,~ID_i,~H_i$ simultaneously to implement it successfully. Adversary might get $Si_3,~ Ui_2$ by side channel attack or by power analysis attack but then he also need $ID_i$ and $H_i$, which is not possible.

#### Privileged insider attack

Since in registration phase user $U_i$ send $ID_i,~Ui_1$ to the server $Sr_j$, where $Ui_1=h(ID_i||Ps_i||H_i)$. So it is not possible for any insider to know the password and biometric of any user because these are protected by hash function *h*(.). It makes proposed scheme secure against privileged insider attack.

#### Impersonation attack

If $$\mathcal {A}$$ wants to act like a legitimate user, he needs to send $$NID_i$$ correctly to the server, where $$NID_i=(Ui_3||Ui_5||ID_i) \oplus T_y (Si_1)=(Ui_3||Ui_5||ID_i \oplus T_y T_s(ID_i)$$. To create $$NID_i$$, $$\mathcal {A}$$ needs $$Si_2)$$, which is protected by $$Ui_1$$ and $$Ui_1=h(ID_i||Ps_i||H_i)$$. So basically $$\mathcal {A}$$ needs $$ID_i,~Ps_i$$ and $$H_i$$ to impersonate user.

Again if $\mathcal {A}$ wants to impersonate server then he need to create $Si_4$ correctly and for this $\mathcal {A}$ needs server’s long term term secret key *s*. So above discussion suggest that proposed scheme is secure against impersonation attack.

#### Reply attack

Since we use different random number *y* for every session in proposed scheme where session key depends upon *y*, with this we also use time stamp, to avoid this type of attacks. For every session, the timestamp is uniquely chosen. The timestamp uniqueness property limits the duplication of log-in messages. This indicates that the proposed system is responsive to replay attacks.

#### Perfect forward secrecy

Even if long term secret key *s* is compromised, suggested scheme is secure. Because to create session key $\mathcal {A}$ needs to compute *x* and for this $\mathcal {A}$ need $ID_i$ which is not possible because we do not send $ID_i$ through public channel.

#### Man in the middle attack

If any adversary $\mathcal {A}$ wants to implement man in the middle attack then firstly $\mathcal {A}$, intercept the massage $Ui_4, ~NID_i,~T_1$, where $Ui_4=T_y(ID_i)$ and $NID_i=(Ui_3||Ui_5||ID_i) \oplus T_y (Si_1)=(Ui_3||Ui_5||ID_i \oplus T_y T_s(ID_i)$. Since $ID_i$ of $U_i$ is hidden in $NID_i$ and it is not practically possible for any adversary to extract $ID_i$ from public channel information therefore adversary fails to forge $Ui_4$. So proposed scheme is secure against man in the middle attack.

#### Stolen card attack

If adversary get access to the smart card of a user and extract $P_i,~Si_3$ and $Ui_2$ from smart card. Even then he could not able to get any meaningful information, that helps $\mathcal {A}$ to breach the security of proposed protocol because all of these are secured by hash function. $\mathcal {A}$ needs $ID_i,~ Ps_i$ and $H_i$ simultaneously get useful information which is not possible.

### Simulation and output using “Automated Validation of Internet Security Protocols and Applications”

**Fig. 6:**
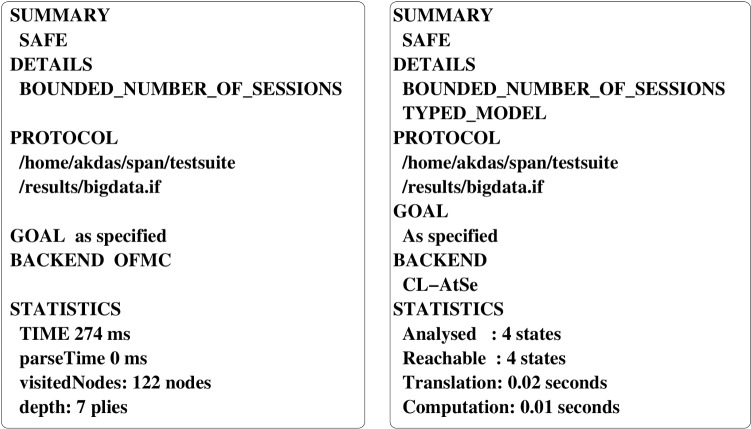
Illustration of output of HLPSL code in OFMC and ATSE modes

In this subsection, we simulate the scheme using “Automated Validation of Internet Security Protocols and Applications” in short AVISPA tool to analyse formal security (man in the middle attack, replay attack) [36]. We have provided essential illustration on the basic output in OFMC and ATSE modes in Fig. [6].

**Fig. 7:**
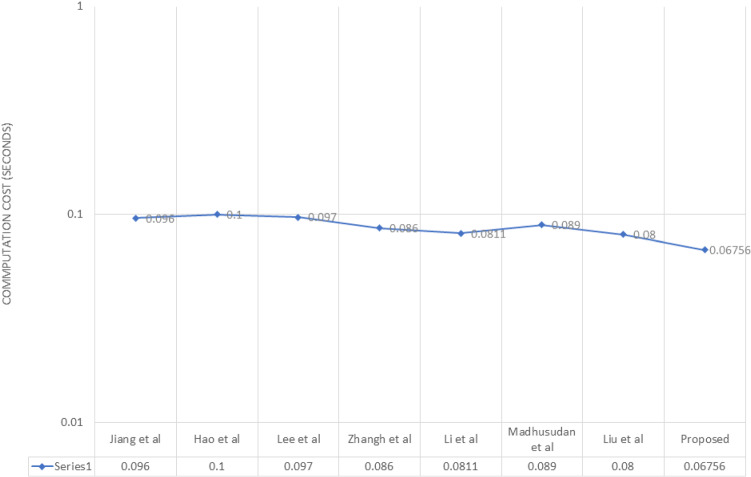
Computation cost comparison

**Fig. 8:**
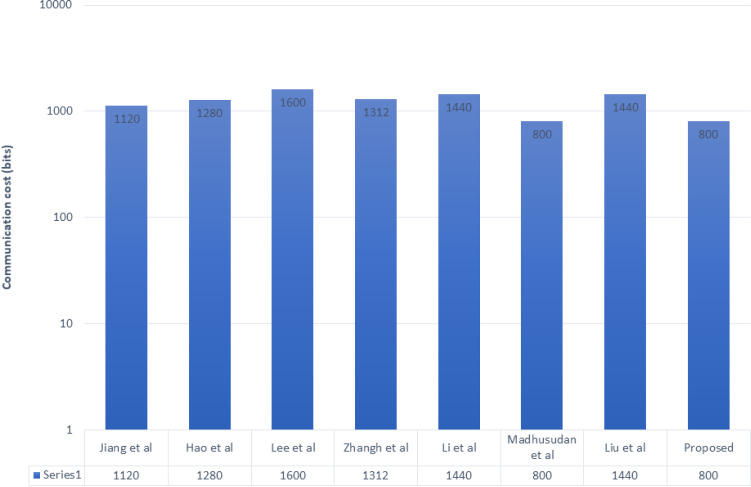
Communication cost comparison

## Performance analysis

In this section, we have analyzed the performance of the proposed protocol and the performance of the proposed protocol has been compared with the related chaotic map based authentication scheme in the Table 4, where the cost of various operations are $h_t \approx 0.0005 s$, $s_{t} \approx 0.0087 s$, $m_t \approx 0.06307 s$, and $c_{t} \approx 0.02102 s$ denote the time for hashing, message encryption under symmetric key, one ordinary multiplication in $Z_p^*$ and chaotic based operation respectively.

**Table 4:** A performance analysis of the proposed protocol with recent chaotic map-based authentication protocols

Schemes	User-computation	Server-computation	Messages
Liu et al.’s [22]	$4h_t + 2 c_t$	$5h_t + 2 c_t$	3
Jiang et al.’s [16]	$2h_t + s_t + c_t$	$2h_t + 2s_t + 3c_t$	2
Hao et al.’s [15]	$$2c_t + 3h_t+ 2s_t$$	$$ 2c_t + 3s_t + 2h_t$$	2
Lee’s [24]	$$2c_t + 7h_t$$	$$ 2c_t + 8h_t$$	2
Zhang et al.’s [25]	$$ 6h_t+2c_t$$	$$ 4h_t+1c_t+2s_t$$	3
Madhusudhan et al’s [20]	$7h_t+2c_t$	$3h_t+2c_t$	2
Li et al.’s [1]	$7h_t+2c_t$	$7h_t+2c_t$	2
Proposed	$4c_t + 4h_t$	$2c_t + 4h_t$	2

We have analysed the performance of chaotic map based authentication protocols [1, 15, 16, 20, 22, 24, 25] with the proposed protocol. As we know our mobile phones has limited storage and random access memory, and internet connectivity is another problem, that all the telecare medicine services runs on limited bandwidth network that is why we need a secure and efficient authentication protocol. Both computation and communication efficiency are very important and these two costs of computations have been compared with existing protocols in the Table 4. The various operation cost estimated via executing an experiment on intel $Pentiums-4$ (1024 MB ram) processor as in [6, 35] with this computation cost described in Fig. 7.

In addition, Liu et al. [22] runs with computation cost $$4h_t + 2 c_t$$ at user side, $$5h_t + 2 c_t$$ at server side, Jiang et al.  [16], at user side runs with computation cost $$2h_t + s_t + c_t$$ at server side use $$2h_t + 2s_t + 3c_t$$, Hao et al.  [15] at user side runs with computation cost $$2c_t + 3h_t+ 2s_t$$, at sever computation cost is $$ 2c_t + 3s_t + 2h_t$$, Lee et al.  [24], at the user side runs with computation cost $$2c_t + 7h_t$$ , at the server side it takes $$ 2c_t + 8h_t$$ , Zhang et al.  [25] runs with computation cost $$ 6h_t+2c_t$$ on the user side, and at server cost is $$ 4h_t+1c_t+2s_t$$ , Madhusudhan et al.  [20] at user side runs with computation cost $$7h_t+2c_t$$, at server side $$3h_t+2c_t$$, Li et al. [1] runs with computation cost $$7h_t+2c_t$$ at the user side, and at server side cost is $$7h_t+2c_t$$, whereas the suggested protocol runs with computation cost $$4c_t + 4h_t$$ at the user side, $$2 c_t + 4h_t$$ at the server side respectively.

In this article, we have considered the cost of communication in the form of hashing, chaotic operation, and time-stamp as 160-bits, and symmetric encryption outputs standard 256 bits, whereas total cost of communication is given in the Fig. 8 and the cost of computation is shown in the Fig. 8.

## Conclusion

This article provides a review on the security of recently proposed chaotic map based authentication protocol. The suggested design is free from most of the existing vulnerabilities such as password-guess, identity-guess, impersonations, replaying of messages, and stolen smart cards attacks and it also gives the idea how a poor verification results vulnerabilities. Furthermore, we have observed that the proposed design fulfills the requirement of session key verification in just two message exchange. In future, we can implement the protocol in vehicular communications, and digital rights management system etc.
